# Using Unannounced Standardized Patients to Assess the Quality of Tuberculosis Care and Antibiotic Prescribing: A Cross-Sectional Study on a Low/Middle-Income Country, Pakistan

**DOI:** 10.3390/antibiotics14020175

**Published:** 2025-02-11

**Authors:** Mingyue Zhao, Ali Hassan Gillani, Hafiz Rashid Hussain, Hafsa Arshad, Muhammad Arshed, Yu Fang

**Affiliations:** 1 Department of Pharmacy Administration and Clinical Pharmacy, School of Pharmacy, Xi’an Jiaotong University, Xi’an 710049, China; mingyue0204@xjtu.edu.cn (M.Z.); hassangillaniali@yahoo.com (A.H.G.); hafsaarshad46@gmail.com (H.A.); 2 Center for Drug Safety and Policy Research, Xian Jiaotong University, Xi’an 710049, China; 3 Shaanxi Centre for Health Reform and Development Research, Xi’an 710061, China; 4 College of Pharmacy, University of Sargodha, Sargodha 40100, Punjab, Pakistan; rashid.hussain@uos.edu.pk; 5 Department of Community Medicine, Baqai Medical College, Baqai Medical University, Karachi 75340, Sindh, Pakistan; drarshedchaudhary@gmail.com; 6 University Institute of Public Health, Faculty of Allied Health Sciences, University of Lahore, Lahore 54590, Punjab, Pakistan

**Keywords:** standardized patients, antibiotic prescribing, tuberculosis, Punjab, Pakistan

## Abstract

**Background:** Pakistan is classified as a high-burden country for tuberculosis, and the prescription of antibiotics and fluoroquinolones complicates the detection and treatment of the disease. The existing literature primarily relies on knowledge questionnaires and prescription analyses, which focus on healthcare providers’ knowledge rather than their actual clinical practices. Therefore, this study aimed to evaluate the quality of tuberculosis care using standardized patients. **Materials and Methods:** We conducted a cross-sectional study, recruiting consenting private healthcare practitioners in four cities in Punjab, Pakistan. Standardized patients were engaged from the general public to simulate four cases: two suspected tuberculosis cases (Case 1 and 2), one confirmed tuberculosis case (Case 3), and one suspected multidrug-resistant tuberculosis case (Case 4). The optimal management in Cases 1 and 2 was referral for sputum testing, chest X-ray, or referral to a public facility for directly observed treatment short-courses without dispensing antibiotics, fluoroquinolones, and steroids. In Case 3, treatment with four anti-TB medications was expected, while Case 4 should have prompted a drug-susceptibility test. Descriptive statistics using SPSS version 23 were employed to analyze disparities in referrals, ideal case management, antibiotic use, steroid administration, and the number of medications prescribed. **Results:** From July 2022 to May 2023, 3321 standardized cases were presented to private healthcare practitioners. Overall, 39.4% of tuberculosis cases were managed optimally, with Case 3 showing the highest rate (56.7%) and Case 4 showing the lowest (19.8%). City-specific analysis revealed that Rawalpindi had the highest management rate (55.8%), while Sialkot had the lowest (30.6%). Antibiotics were most frequently prescribed in Case 1 and least prescribed in Case 4, with a similar pattern for fluoroquinolones. Anti-TB medications were also prescribed in naïve and suspected tuberculosis cases (8.3% in Case 1 and 10.8% in Case 2). **Conclusions:** The quality of tuberculosis management in actual practice is suboptimal among healthcare providers in Pakistan. Furthermore, the over-prescription of antibiotics, fluoroquinolones, and anti-TB drugs presents a significant risk for the development of drug-resistant tuberculosis.

## 1. Introduction

Pakistan ranks fifth globally in tuberculosis (TB) cases, contributing to 5.7% of the global TB burden and 70% of the regional burden [1,2]. In 2021, TB was the second leading cause of death from a communicable disease, responsible for nearly 1.6 million fatalities worldwide [3]. The misuse of antibiotics and fluoroquinolones can contribute to the development of multidrug-resistant tuberculosis (MDR-TB), which has become an emerging health crisis in developing countries [4,5]. In Pakistan, the TB burden is exacerbated by uneven healthcare services, diagnostic delays, improper medication regimens, treatment discontinuation, incomplete treatment courses, inadequate follow-up, and insufficient social support [4,6,7]. This issue is not unique to Pakistan but is also seen in other countries in the region, such as India, where the fragmented healthcare system (HCS) and an unregulated private sector have been linked to the growing TB burden [8,9,10].

Concerns about the quality of TB care in Pakistan have arisen from existing data on TB management [5,6,7,11]. A prior study indicated a lack of understanding of TB among private sector practitioners [12], while another study found that screening for childhood TB was adequately documented by private sector physicians [13]. The variation in these results is likely due to the reliance on surveys, which are susceptible to recall bias. Evidence suggests that TB patients often navigate complex healthcare pathways, consulting multiple healthcare providers (HCPs) before receiving a diagnosis and treatment [14,15]. TB patients frequently seek care from private HCPs, independent practitioners, clinics, non-governmental organizations, and community pharmacies.

Previous evaluations of TB care quality were based on patient recall surveys, knowledge questionnaires, and the analysis of prescriptions and medical records, which did not accurately reflect actual clinical practices [16,17,18,19]. Consequently, standardized patients (SPs)—individuals trained to simulate patients in medical scenarios—are increasingly being used to assess the quality of medical care in both developed and developing countries [17,18,19,20,21,22]. Data obtained from SPs offer a more accurate evaluation of HCP practices that is free from observation bias and less prone to recall bias compared to patient exit interviews [23]. Furthermore, SPs provide a more comprehensive view of healthcare practices than medical records alone and can help estimate case detection rates due to the intentional design of specific illnesses. In Pakistan, there is a significant gap in studies using advanced methods to observe real-world practices in private clinics, although limited studies have investigated dispensing practices in community pharmacies [4,24,25,26,27]. This study aims to assess real-world practices in TB care using the SP methodology.

## 2. Results

### 2.1. Detection Rates of Standardized Patients

The overall detection rate of the SPs was 3.6%, which was below the accepted threshold. However, detection rates varied by case: Case 1 had a detection rate of 0%, Case 2 had 2.3%, Case 3 had 2.4%, and Case 4 had 10.5%. The details are given in Table 1.

### 2.2. City-Wise Ideal Case Management

Out of 1058 Case 1 scenarios, 488 (46.1%) were completed in Lahore, 307 (31.3%) in Rawalpindi, 163 (15.4%) in Sialkot, and 100 (9.4%) in Faisalabad. Of the 1058 Case 1 encounters, 455 (43.0%) were referred to a specialist or directly observed treatment short-course (DOTS) facility, and 377 encounters were optimally managed. In Lahore, 35.5% (173) were referred, and 28.7% (140) were ideally managed. In Rawalpindi, 61.2% of encounters were referred to DOTS or a specialist, while 32.9% of Sialkot encounters were similarly referred. Overall, 32.9% of cases in Lahore, 30.1% in Rawalpindi, 15.3% in Sialkot, and 33.0% in Faisalabad received antibiotics. The most concerning finding was that 4.7%, 3.7%, and 0.0% of Case 1 visits in Lahore, Sialkot, and Faisalabad, respectively, received anti-TB medications.

We observed 992 Case 2 encounters across the four cities: Lahore (44.8%), Rawalpindi (29.9%), and Sialkot (17.1%). Of these cases, 62.2% were well managed in Lahore, 54.5% in Rawalpindi, and 15% in Faisalabad. Antibiotics were administered in 11.9% of Case 2 encounters, fluoroquinolones in 15.9%, and steroids in 17.6%.

The optimal case management in Case 3 differed from the previous two. A total of 958 interactions were observed in Case 3. Of these, 456 (47.6%) were referred to a specialist or DOTS treatment, and 544 (56.7%) were optimally managed. The proportion of fluoroquinolones to antibiotics decreased compared to the previous cases (18.0% antibiotics, 22.5% fluoroquinolones). However, the percentage of anti-TB medications rose to 36.1%. Notably, none of the cases were prescribed drugs from Schedule G (Opioids) of the Pakistani drug category. All relevant data are available in Table 2.

### 2.3. Number of Medicines Prescribed in Different Cities

Regarding the number of medications dispensed, in Case 1, the most commonly prescribed drugs were over-the-counter (OTC) medications (50.5%), followed by antibiotics [306/1058 (28.9%)] and fluoroquinolones (22.8%). Among antibiotics, the most commonly prescribed were amoxicillin + clavulanic acid (11.0%) and amoxicillin (9.5%). A total of 241 individuals were prescribed fluoroquinolones (such as levofloxacin, ciprofloxacin, and ofloxacin), while 14.5% were given steroids like prednisolone and betamethasone. This pattern changed in Case 4, where the highest proportion of medications prescribed were anti-TB drugs (63.4%), followed by OTC medications (36.6%). Among the OTC medications, in Case 1, the most commonly given was Cetirizine followed by Diphenhydramine, and the second most commonly prescribed in Case 4 was Panadol followed by ibuprofen. Detailed data can be found in Table 2.

### 2.4. Gender-Wise Differences in Ideal Case Management

There was variation in referrals and ideal case management based on the gender of the HCPs. However, referrals did not show significant gender differences in Case 1 (44.4% males vs. 40.9% females), Case 2 (67.2% males vs. 74.9% females), or Case 4 (11.9% males vs. 11.9% females). Similarly, there were no significant gender differences in ideal case management in Case 1 (36.5% males vs. 34.4% females), Case 2 (57.5% males vs. 53.6% females), and Case 4 (20.2% males vs. 19.4% females). However, a significant gender difference was observed in the referral (68.9% males vs. 52.3% females) and ideal management (68.9% males vs. 52.3% females) in Case 3 (Details are given in Table 3).

### 2.5. Number of Drugs Prescribed by Different Genders

Males in our study prescribed antibiotics more frequently than females in Cases 1, 2, and 4, whereas females prescribed more antibiotics in Case 3. In contrast, the prescribing of fluoroquinolones was significantly higher among females in Cases 1, 2, and 3, with an insignificant difference in Case 4. The most frequently prescribed medications by all providers were OTC medications. Among females, 36.4% (485/1332) prescribed OTC medications, while 34.6% (688/1989) of males prescribed OTC medications to SPs. Detailed data can be found in Table 3.

**Figure 1:**
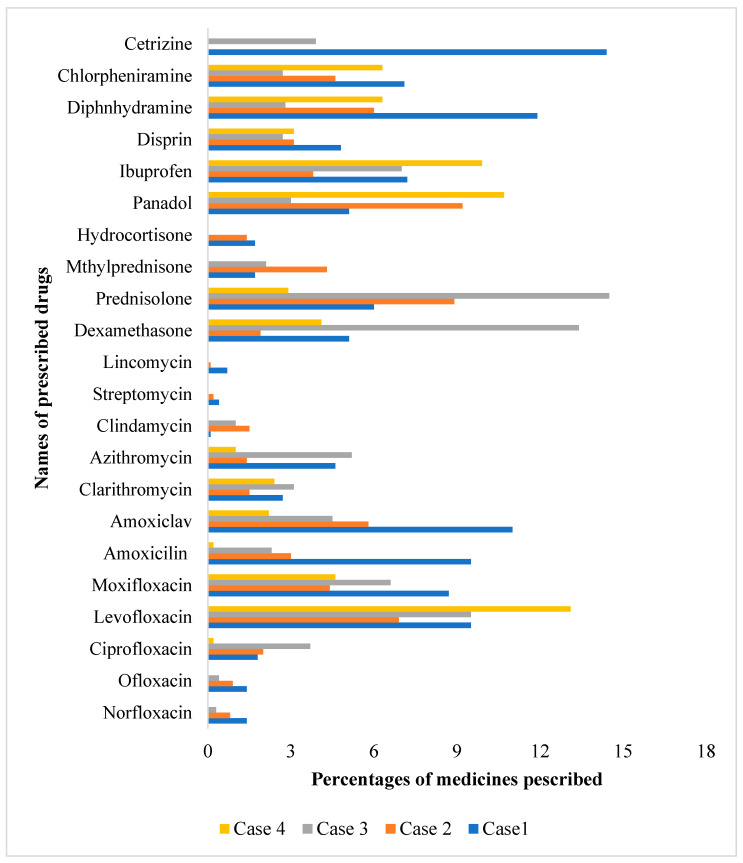
Percentage of each medicine prescribed in each case type.

In an overall trend, it can also be seen that the highest number of drugs given was cetirizine in Case 1, followed by prednisolone and steroids in Case 3. In the case of Fluoroquinolones, it is seen that levofloxacin was given in the highest quantity in Case 4 and amoxiclav in Case 1. Details can be seen in Figure 1.

**Figure 2:**
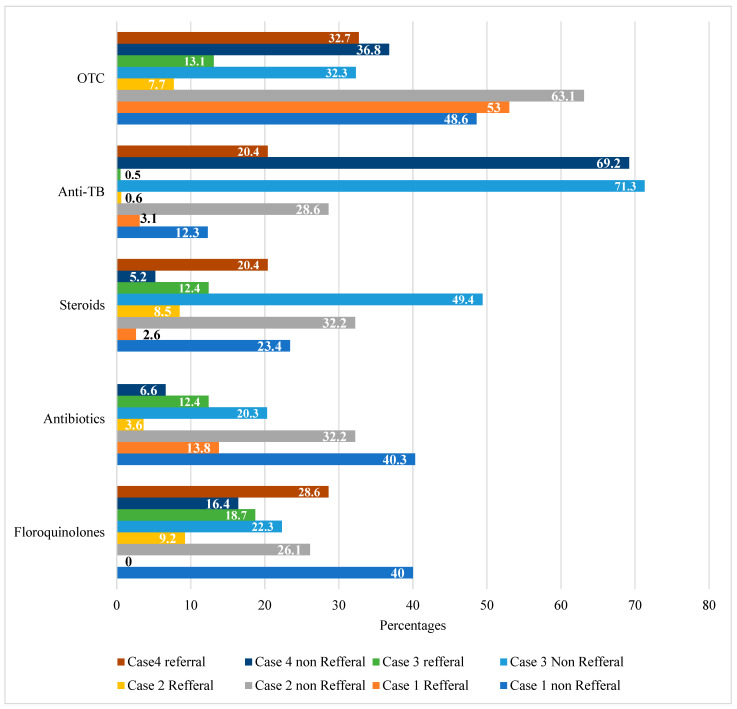
Representation of all cases (referred and non-referred) and drugs prescribed in them.

As shown in Figure 2, each panel set demonstrates the percentages of drugs used in different cases. The first panel set shows the usage of fluoroquinolones in each case (referred and non-referred cases). Similarly, the second, third, fourth, and fifth panel group represents the usage of antibiotics, steroids, and anti-TB and OTC medicines in all cases (referred and non-referred), respectively. All referral and non-referral cases in Cases 1–4 are presented in percentages. According to the results in Figure 2, we can see that the highest amount of antibiotics was prescribed in Case 1 (non-referral) and the least prescribed for referred patients in Case 2, but none was prescribed for the referred cases in Case 4.

## 3. Discussion

Pakistan is ranked fifth among the 22 countries with a high burden of TB [1,2,7]. The nation has made significant progress in managing MDR-TB, particularly through the implementation of DOTS and the programmatic management of MDR-TB. Despite these efforts over the past decades, Pakistan continues to face substantial challenges in managing and eliminating MDR-TB, with both morbidity and mortality rates rising [15,28,29,30]. The World Health Organization (WHO) launched the “End TB” campaign, which emphasizes patient-centered treatment and the involvement of all HCPs [31]. In 2009, the WHO introduced a TB infection control (TBIC) policy to reduce TB transmission, which was subsequently integrated into Pakistan’s national TBIC plan [32,33].

Given the significant role of the private sector in the provision of healthcare in Pakistan, it is crucial to understand how private practitioners manage TB and identify the factors contributing to suboptimal care [34]. This knowledge is essential for the development of effective public–private mix interventions. This study represents the first evaluation of TB care quality in Pakistan using the SP technique. Historically, TB treatment has not been assessed in private clinics; however, research involving SPs was conducted by us in pharmacies, which highlighted poor management practices for TB [4]. This study demonstrated that the SP methodology is acceptable in Pakistan to evaluate TB care, with detection rates comparable to those in other SP studies, including those from India [17,35,36]. The SP approach can be further refined by extending the study period. The findings are credible and provide a valid comparison across HCPs of various countries [37,38].

Our primary findings revealed an increase in ideal case management from uncertain to confirmed diagnoses (Cases 1–3) but a decline in management for MDR-TB cases (Case 4). Additionally, there was significant variation in ideal case management across all four cases and cities in Punjab, Pakistan. These findings are consistent with a similar study conducted in India [39]. The suboptimal treatment quality was characterized by the insufficient use of appropriate diagnostic tests and the frequent prescription of unnecessary medications, including antibiotics, contraindicated fluoroquinolones, and steroids. TB-specific treatment increased with diagnostic certainty in Rawalpindi and Faisalabad but remained stagnant in Lahore and Sialkot. This finding was unexpected, as previous studies have suggested that increased diagnostic certainty improves the quality of care in pharmacies in Pakistan [4]. This phenomenon has also been observed in other countries [39,40]. Providers managed the SPs in a highly variable manner without a standardized approach. Data suggest that excessive patient loads may contribute to the low quality of care. Among 3321 encounters, only 9.4% had no other patients waiting, 60.5% had at least one patient in line, and 30.1% had two or more patients waiting. This contrasts with findings from other SP studies in other healthcare settings [39,40,41]. We also identified two major reasons for the observed behavior: (a) difficulties in diagnosing TB and (b) private HCPs violating established standards for financial gain. Increasing diagnostic certainty positively impacted care quality, supporting the notion that inadequate diagnostic skills contribute to suboptimal care. However, the lack of diagnostic confidence was not the only factor contributing to low-quality care. The increase in diagnostic certainty improved accurate case management but negatively affected the prescription of antibiotics and fluoroquinolones, which undermines the validity of this effect. Previous research has also indicated that promotional activities and financial incentives lead to increased prescription rates for antibiotics [42,43]. A study by Kwan et al. highlighted significant deficiencies in the management of TB patients in Mumbai and Patna [39]. Our findings emphasize the disparities in TB care quality across cities, underscoring the need for collaboration with the private sector to improve TB care.

Our study also revealed that one in five individuals were prescribed antibiotics, and 18.1% received fluoroquinolones. These medications may delay TB diagnosis, which is concerning [44]. The current recommendations for treating other respiratory diseases may modify the outcome of TB management, i.e., the Infectious Diseases Society of America and the American Thoracic Society advocate for the use of respiratory fluoroquinolones in managing adult community-acquired pneumonia with co-morbidities [45]. The empirical treatment of community-acquired pneumonia with fluoroquinolones poses significant concerns regarding the delayed detection and treatment of TB and the emergence of fluoroquinolone-resistant Mycobacterium TB [46]. Multiple case studies indicate that the use of fluoroquinolones can postpone the identification of TB and contribute to the development of fluoroquinolone-resistant Mycobacterium TB [47,48]. Evidence from a trial suggests that for individuals in the group receiving fluoroquinolones, the average duration of healthcare delay was 19.44 days, much greater than that in the non-fluoroquinolone group; however, the mean difference in antibiotic delay was not statistically significant (15.69 days) [44]. The widespread use of antibiotics and steroids for respiratory conditions has contributed to an increase in community-acquired infections. The overuse of fluoroquinolones has also led to an increase in diarrheal diseases and the emergence of resistant Gram-negative enteric bacteria, particularly in Pakistan and India [49]. There are also city-wise differences in the prescriptions of fluoroquinolones; in our study, these variations were similar to those observed in previous situations in Pakistan and India [4,39]. Furthermore, our data revealed that 8.3% of patients in Case 1 and 10.2% in Case 2 received first-line anti-TB medications. This raises concerns about the prescribing practices for anti-TB drugs by private doctors in major urban centers in Pakistan. If these practices persist, they may contribute to the rise in MDR-TB. These findings are in contrast to those from India, where no patients received anti-TB medications [39], but align with earlier findings from Pakistan [4]. Further research is needed to understand why HCPs are prescribing TB medications inappropriately, as these prescribers should follow national and international guidelines. Overall, 14.5% of cases had steroids prescribed, which was especially high in Case 3 in three cities (Rawalpindi, Lahore, and Sialkot) and Case 4 in Faisalabad. These results were in line with the previous studies from Pakistan where there were significantly fewer steroids dispensed in confirmed TB cases across all the cities [4]. Similarly, a study from India also reported that there is a disparity in the prescription of steroids for all four cases across two cities [39]. These results are somewhat satisfactory as prescribing steroids in different TB cases may worsen the situation, mask the symptoms, and delay the detection.

Our study has several strengths. First, we sampled a large number of private HCPs across four Pakistani cities, providing accurate estimates of provider behavior at the city level. Second, by using unannounced SPs, we captured actual provider behavior, rather than relying on self-reported knowledge or practices. The SP method better reflects real patient–provider interactions than other existing methods used to measure the quality of care. Third, by developing four distinct SP case presentations, we studied how providers handled various stages of TB and varying levels of diagnostic certainty. Lastly, by analyzing outcomes by city and the gender of providers, we assessed key sources of variation in care quality.

However, our study also has limitations. Firstly, our SPs did not exhibit physical signs (e.g., crackles) that could be detected through chest auscultation, which may have misled providers due to the absence of physical findings. Secondly, the SP method is better suited for one-time patient interactions rather than multiple visits to the same provider or interactions with known patients. We cannot ascertain how a provider would manage a patient after receiving a chest X-ray (CXR) that they had recommended, as opposed to a CXR ordered by another doctor. A design involving repeated SP visits could offer additional insights, though it would be challenging to implement. This study did not evaluate the long-term outcomes of provider decisions, such as adherence to treatment or patient recovery, which limits its ability to assess the ultimate impact of the observed care practices on TB management. Thirdly, we did not observe how patients actually choose HCPs. Factors such as qualifications, geography, personal relationships, price, reputation, and other unobserved signals of quality influence patient selection, limiting the generalizability of our findings to actual TB patients. Future studies should explore this aspect. While not a limitation per se, we note that our definition of correct case management follows national and international TB treatment standards. These definitions allow for comparability across studies and disciplines, and we analyzed the data using a pre-specified protocol. Despite these limitations, our results align with findings from Indian studies that have shown low adherence to recommended standards and considerable variability in provider practices [8,20,34,35,39,40].

## 4. Materials and Methods

### 4.1. Recruitment, Training, and Detection Rate of Standardized Patients (SPs)

We recruited SPs from the general public, selecting ordinary individuals as data collectors to enhance the realism of the encounters. The use of specialized performers was deliberately avoided to minimize complexity. Participants were recruited through targeted advertisements aimed at the general population, excluding professional actors. Individuals who appeared healthy were chosen as SPs. Applicants contacted the principal investigator (PI), who conducted screening interviews. A total of 32 SPs were recruited to participate in the training sessions, which spanned two months, from January to February 2022. During the first month, they received intensive training covering various elements and case presentations. Following the training, participants were assigned cases based on their performance in dry runs. All SPs were instructed on safety measures to prevent exposure to harmful medical procedures, such as intrusive tests, injections, or the consumption of medications. We selected 23 SPs (7 in Case 1, 6 in Case 2, 5 in Case 3, and 5 in Case 4), including 16 males and 7 females. Three SPs had basic education, eleven were undergraduates, and nine were postgraduates. Eighteen had a household income below PKR 50,000, while five had incomes above PKR 50,000. A city head, who was a lecturer/assistant professor at a local institution, was designated for each location, and SPs were instructed to report to them for assistance. The PI regularly visited several cities to receive updates from the city heads and SPs.

The risk of SP detection had not been previously assessed; however, we reduced this risk by selecting SPs unfamiliar to HCPs. Before starting this study, we designed a detection survey and validated the SP technique by collecting data from 44 private HCPs in two locations in Punjab, Pakistan. These HCPs were informed and consented to being visited by an individual posing as a patient over the next two months. If they suspected any patient was inauthentic, they were instructed to document the patient’s name and visit date. The providers were assigned randomly. Following 168 successful encounters (Cases 1–4) with the 44 HCPs, the detection survey was conducted according to established procedures [50]. Conversations were recorded by randomly chosen SPs using mobile recorders to assess the accuracy of their recollections, as evaluated through a predefined recall questionnaire [51]. Three to four weeks after the SP visits, the PI conducted follow-up visits to the physicians to determine whether any patients were suspected of being fraudulent. If any suspicions arose, the physicians were asked to describe the symptoms, age, and sex of the suspected SPs. The detection rate was calculated by dividing the number of SPs identified by the providers (true positives) by the total number of SP encounters. These providers who have been part of the detection survey were not included in the main study. These are only limited to detection surveys, and, afterward, we omitted them. Detection rates in similar SP studies are typically around 5%.

### 4.2. Case Development and Presentation

We developed four TB tracer scenarios based on global, regional, and national guidelines [52,53,54]. While several standards exist, we focused on the critical aspects of TB diagnosis and treatment and assisted in creating case-specific checklists of required and recommended therapies. These case scripts were developed in collaboration with social scientists, the PI, and recruited SPs to ensure clarity in diagnosis and presentation. For example, in Case 1, relevant inquiries revealed that the patient had a cough lasting 2–3 weeks, produced sputum, experienced fever with night sweats, and had lost appetite and weight. According to our standards, this information should prompt the clinician to consider TB and initiate appropriate microbiological tests or refer the patient for TB evaluation. All scripts were written in English. The four TB case scenarios were designed to assess the quality of care provided for TB by selected providers, highlighting variations in care quality. Each case’s clinical presentation and social context were reviewed and approved by a technical advisory panel consisting of physicians, economists, anthropologists, and TB specialists. The cases included are as follows:

- **Case 1: Naïve TB Suspect**
  *Case Description:* A typical case of probable TB with a 2–3-week cough and fever.
  *Case Exposition:* The patient initiates the conversation with “Doctor, I have a persistent cough and fever that is not improving.”
  *Anticipated Outcome:* Suggestion for sputum analysis, chest X-ray, or referral to a public DOTS facility or a qualified provider.
- **Case 2: TB Suspect with Abnormal Chest X-Ray**
  *Case Description:* A patient presenting with a 2–3-week history of cough and fever, an abnormal chest X-ray, and a prior prescription for broad-spectrum antibiotics with no improvement.
  *Case Exposition:* The SP presents a digital chest X-ray and a blister pack of amoxicillin, saying “Doctor, I’ve had a cough and fever for weeks, but my condition is not improving despite seeing a physician and taking the prescribed medication.”
  *Anticipated Outcome:* Recommendation for sputum analysis, further chest radiography, or referral to a public DOTS facility or a skilled practitioner.
- **Case 3: Confirmed TB Case**
  *Case Description:* A persistent cough with a positive sputum smear test for TB from a public health center. The SP holds the sputum test report.
  *Case Exposition:* The patient says: “I’ve been coughing for a long time and have a fever. I went to the government hospital where I got medication and had sputum testing.”
  *Anticipated Outcome:* Referral to a public DOTS center, a qualified private provider, or, if a private provider is qualified, the initiation of first-line anti-TB therapy (isoniazid, rifampicin, pyrazinamide, and ethambutol).
- **Case 4: Multidrug-Resistant (MDR) TB Suspect**
  *Case Description:* A patient with 1 month of cough and fever, and a history of incomplete TB treatment, suggesting the possibility of MDR-TB.
  *Case Exposition:* The SP says: “Doctor, I’m experiencing a bad cough. I was treated for TB at the government hospital a year ago, and it got better, but now I have a cough again. I went back to the same hospital, and they did a sputum test.”
  *Anticipated Outcome:* Recommendation for drug susceptibility testing (culture, line probe assay, or Xpert MTB/RIF) or referral to a public DOTS center or a specialist.

### 4.3. Study Design and Participants

The target group of this study consisted of practicing physicians in the private sector across four cities in Punjab, Pakistan: Sialkot, Lahore, Rawalpindi, and Faisalabad, from July 2022 to May 2023. We chose private sector because, like other countries, it serves more than two-thirds of the population seeking healthcare services in Pakistan [32,55,56,57]. Also, in Pakistan, due to the subpar state of public division facilities, the private sector remains the preferred choice, with over 90% of people accessing it for TB care [57]. The physician-to-patient ratio in Pakistan is 1.1 per 1000 patients, which is below standard levels, with the majority of physicians working in private practice [58]. There is no centralized database in Pakistan that provides detailed information on the private clinical practice settings of these physicians.

Before data collection, the premise addresses were obtained through a comprehensive road-to-road mapping process, with assistance from the city head and local pharmacists. The cities were divided into multiple sectors, and a few data collectors were assigned the task of identifying as many physicians as possible. Although this method limited the generalizability of the data, it has been employed in previous studies [8,16,20]. Upon reaching the practice locations, address, practice specialty, and registration number of each prescriber were recorded to maintain a detailed record list for SPs and case presentations. Physicians eligible for the SP study were limited to those recognized for treating adult outpatients with respiratory symptoms in the private health sector. This category primarily encompasses most primary care physicians such as basic MBBS graduates, general practitioners, and specialists in ENT, pulmonology, oncology, and thoracic surgery, while those from other specialties such as orthopedists, gynecologists, ophthalmologists, cardiologists, and pediatricians were excluded. Physicians engaged in academic work, on leave, or holding administrative positions were also excluded. The route mapping and listing process was completed within one month (March–April 2022). Each case was presented by a single SP, who represented only that case. For example, SPs X and Y were the sole presenters in Case 1. We ensured that each case was presented by both male and female SPs in every city to minimize potential confounding factors. The HCPs were targeted at a particular time point to complete the interaction; however, if it was not possible due to overload or any other problem, we decided to plan a second visit.

### 4.4. Exit Questionnaire

The exit questionnaire included the following sections:

- Cover Page: This included the form number, facility ID, physician’s ID, private clinic details, visit details, date of interaction, and the start and end times of the consultation, along with other relevant information regarding the visit.
- Case-Specific Historical Inquiries: Physicians were required to ask case-specific historical questions, with another option for additional details.
- Clinical or Physical Exams: Any exams conducted (often not case-specific) were recorded, with an “other” option to specify additional tests.
- Diagnostic Tests: Diagnostic tests requested by the physician were listed, with an “other” option to include any unspecified tests.
- Diagnosis, Referral, and Follow-up: Information about the diagnosis provided, any referrals made (with specific details), and “return to provider” instructions (with relevant facts).
- Prescribed Medications: This section recorded the prescribed medications, including their price, quantity, point of acquisition, and Anatomical Therapeutic Chemical classification code where feasible.
- Consultation Fees: Detailed fees for consultations, laboratory tests, and pharmaceuticals, with a breakdown where possible, or summarized when not.

The full questionnaire is included as Supplementary Material. All SPs paid providers at their standard consultation rate. We evaluated the quality of care by assessing HCPs’ adherence to case-specific checklists of recommended practices, the appropriateness of treatments provided, and the use of unnecessary or inappropriate therapies (e.g., steroids). These checklists were based on both international and national guidelines [52,53]. Additionally, we collected data on the time and costs associated with each consultation and the medications prescribed. The expenses were calculated using 2023 rates in PKR and included fees paid to the provider during the consultation and the cost of medications, excluding laboratory tests and other operations that were not performed by the HCPs. The questionnaire was completed within half an hour of the interaction by another SP accompanying the SP who presented the cases.

### 4.5. Data Analysis

After data collection, four pharmacists in the individual cities documented a list of labeled medications and prescriptions provided by HCPs to SPs. These medications were then categorized into anti-TB medications, antibiotics (excluding TB drugs), and steroids. Fluoroquinolones were classified separately as a distinct category of antibiotics due to their potential to obscure underlying TB. The pharmacists were also tasked with identifying any loose, unlabeled tablets administered to the SPs. Based on their evaluations, it was determined whether the medications contained at least one antibiotic.

A key concern regarding the SP approach is that the individuals presenting the cases were not actually ill, which could potentially affect the authenticity of their clinical presentations, despite receiving training. We computed the ratio for our primary outcome, which includes interactions leading to optimal case management, and those resulting in the administration of antibiotics, fluoroquinolones, and steroids. Logistic regression was used to assess differences in clinical care procedures and case management among SPs based on gender and location. Results were presented as odds ratios (ORs) for gender. All data were comprehensively analyzed using IBM Chicago Illinoi SPSS version 23.0.

### 4.6. Ethical Approval

The research was conducted according to the requirements of Declaration of Helsinki and we secured ethical approval from the Medical Research Ethics Committee of Xi’an Jiaotong University, China (XJTSP 11-2022), and the Research Ethical Review Board Committee of The Superior University, Lahore (ERB-SP 2022). Since the objective of our study was to understand the unfettered interaction between provider and patients, seeking informed consent from a provider can jeopardize the scientific validity of this study by influencing the decision of providers to take part (creating selection bias) or influencing their behavior (Hawthorne effect) if they think SP visits are imminent. Therefore, both Institutions gave a waiver to forgo obtaining informed consent from providers to mitigate these two effects.

## 5. Conclusions

Optimal care for TB patients is more effective when there is diagnostic certainty, whereas care quality decreases when TB symptoms are ambiguous. The excessive use of antibiotics and fluoroquinolones was noted in all cases, potentially hindering timely diagnosis. Notably, physicians prescribed anti-TB medications to both naive and suspected TB cases, raising significant concerns about their prescribing practices. However, no medications from prohibited categories (Opioids) were administered. TB management in Pakistan remains inadequate, and our findings underscore the urgent need for programs that actively engage clinicians in TB control and improve antimicrobial stewardship practices.

## Figures and Tables

**Table 1:** Case-wise detection rate of standardized patients across different cities.

Lahore				Rawalpindi			
Case 1		Case 2		Case 1		Case 2	
Presented	Detected	Presented	Detected	Presented	Detected	Presented	Detected
21	0 (0.0%)	23	0 (0.0%)	22	0 (0.0%)	21	1 (4.7%)
Case3		Case 4		Case 3		Case4	
Presented	Detected	Presented	Detected	Presented	Detected	Presented	Detected
19	1 (5.3%)	17	2 (11.7%)	22	0 (0.0%)	21	2 (9.5%)

**Table 2:** City-wise management of all 4 cases in Punjab, Pakistan.

**Scenario**	Case 1					Case 2					Case 3					Case 4				
	**Rawalpindi**	Lahore	Sialkot	Faisalabad	*p*	Rawalpindi	Lahore	Sialkot	Faisalabad	*p*	Rawalpindi	Lahore	Sialkot	Faisalabad	*p*	Rawalpindi	Lahore	Sialkot	Faisalabad	*p*
No of interactions	307	488	163	100	0.000	297	445	170	80		160	446	170	82		173	142	70	28	
Referrals	188 (61.2%)	173 (35.5)	52 (32.9)	42 (42.0)	0.000	214 (72.1%)	314 (70.6%)	152 (89.4%)	18 (22.5%)	0.000	61 (38.1%)	280 (62.8%)	56 (32.9%)	59 (72.0%)	0.000	8 (4.6%)	22 (15.5%)	9 (12.9%)	10 (35.7%)	0.000
Ideal case management	163 (53.1%)	140 (28.7)	32 (19.6)	42 (42.0)	0.000	162 (54.5%)	277 (62.2%)	104 (61.2%)	12 (15%)	0.000	146 (92.1%)	277 (62.1%)	49 (28.8%)	72 (75.6%)	0.000	41 (23.7%)	22 (15.5%)	9 (12.9%)	10 (35.7%)	0.02
Drugs																				
Antibiotics	101 (32.9%)	147 (30.1%)	25 (15.3%)	33 (33.0%)	0.000	0 (0.0%)	69 (15.5%)	8 (4.7%)	36 (45.0%)	0.000	50 (31.2%)	22 (4.9%)	65 (38.2%)	36 (31.7%)	0.000	6 (3.5%)	8 (5.6%)	0 (0.0)	10 (35.7%)	0.000
Fluoroquinolones	21 (6.8%)	19 (18.4)	102 (63.2%)	27 (27.0%)	0.000	41 (13.8%)	68 (15.3%)	47 (27.6%)	2 (2.5%)	0.000	0 (0.0%)	147 (33.0%)	69 (40.6%)	0 (0.0%)	0.000	17 (9.8%)	34 (23.9%)	16 (22.9%)	7 (25.0%)	0.000
Steroids	52 (16.9%)	85 (17.4)	8 (4.9%)	8 (8.0)	0.000	58 (19.5)	74 (16.6%)	0 (0.00)	24 (30%)	0.000	63 (39.4%)	127 (28.5%)	115 (67.6%)	12 (14.6%)	0.000	12 (6.9%)	7 (4.9%)	5 (7.1%)	5 (17.9%)	0.112
Anti-TB	59 (19.2%)	23 (4.7%)	6 (3.7%)	0 (0.0%)	0.000	55 (18.5%)	34 (7.6%)	7 (4.1%)	11 (13.8%)	0.000	99 (61.9%)	159 (35.7%)	64 (37.6%)	23 (28.0%)	0.000	60 (34.7%)	120 (84.5%)	68 (97.1%)	14 (50.0%)	0.000
OTC	175 (57.0%)	258 (52.9%)	42 (25.8%)	59 (59.0%)	0.000	84 (28.3)	112 (25.2)	11 (6.5)	55 (68.8%)	0.000	92 (57.5)	53 (11.9%)	53 (31.2)	29 (35.4%)	0.000	17 (9.8%)	53 (37.3%)	59 (84.3%)	21 (75%)	0.000
Schedule G (Opioids)	0 (0.0%)	0 (0.0%)	0 (0.0%)	0 (0.0%)	0.99	0	0	0	0	0.999	0	0	0	0	0.99					

**Table 3:** Providers’ gender-wise management of all 4 cases in Punjab, Pakistan.

Scenario	Case 1		OR (CI)	*p*	Case2		OR (CI)	*p*	Case 3		OR (CI)	*p*	Case 4		OR	*p*
	Male	Female			Male	Female			Male	Female			Male	Female		
No of interactions	633	425			589	403			514	344			253	160		
Referrals	281 (44.4%)	174 (40.9%)	1.138 (0.885–1.464)	0.313	396 (67.2%)	302 (74.9%)	1.072 (0.808–1.423)	0.629	290 (56.4%)	166 (48.3%)	0.720 (0.548–0.947)	0.019	30 (11.9)	19 (11.9)	1.002 (0.543–1.847	0.996
Ideal case management	231 (36.5%)	146 (34.4%)	1.387 (1.071–1.797)	0.013	339 (57.5)	216 (53.6)	0.941 (0.727–1.220)	0.648	354 (68.9%)	180 (52.3%)	0.496 (0.374–0.658)	0.000	51 (20.2%)	31 (19.4%)	0.846 (0.576–1.566)	0.846
Drugs																
Antibiotics	198 (31.3%)	108 (25.4)		0.039	76 (12.9%)	37 (9.2%)		0.070	79 (15.4%)	84 (24.4%)		0.001	19 (7.5%)	5 (3.1%)		0.061
Fluoroquinolones	110 (17.4%)	131 (30.8%)		0.000	76 (12.9%)	82 (20.3%)		0.005	112 (21.8%)	104 (30.5%)		0.005	43 (17.0%)	31 (19.4%)		0.539
Steroids	103 (16.3%)	50 (11.1%)		0.051	87 (14.8%)	69 (17.1%)		0.318	158 (30.7%)	159 (46.2%)		0.000	21 (8.3)	8 (5.0%)		0.21
Anti-TB	55 (8.7)	33 (7.8%)		0.594	70 (11.9%)	37 (9.2%)		0.18	204 (39.7%)	141 (41.0%)		0.704	162 (64.0%)	100 (62.5%)		0.753
OTC	327 (51.7%)	207 (48.7%)		0.346	151 (25.6)	111 (27.5)		0.531	135 (26.3%)	92 (26.7%)		0.804	75 (29.6%)	75 (46.9%)		0.000

Note: Ideal case management and referrals were analyzed by using binary logistic regression (males as references) and drugs prescribed via chi-square analysis.

## Data Availability

The raw data supporting the conclusions of this article will be made available by the authors upon request.

## Supplementary Materials

The following supporting information can be downloaded at: https://www.mdpi.com/article/10.3390/antibiotics14020175/s1. SP TRAINING.
