# Supplementary material for: Using Unannounced Standardized Patients to Assess the Quality of Tuberculosis Care and Antibiotic Prescribing: A Cross-Sectional Study on a Low/Middle-Income Country, Pakistan
Source: Antibiotics (Basel). 2025 Feb 11;14(2):175. doi: 10.3390/antibiotics14020175 (PMC11852056; doi:10.3390/antibiotics14020175)
Supplement: Supplementary file 1 [file antibiotics-14-00175-s001.zip › antibiotics-3436465-supplementary.pdf]

SP TRAINING

Training schedule

Previous research indicated that a three-week duration is sufficient for trainees to remember and internalize scripts, retain exit interviews, contextualize their characters and the attributes of the condition, do mock interviews, and execute dry runs. Considering the variability of circumstances and the occasional unavailability of trainers, healthcare professionals (HCPs), and standardized patients (SPs), we finish the process within two months, allocating 20 days for each specific phase (Phase 1-3).

Phase 1 (Day 1 to Day 20). We enhanced the script narratives and presented the script structure and exit questionnaire to the SPs. We accomplished this through script and story development, group reenactments, and the implementation of exit surveys for the standardized patients.

Phase 2 (Days 21 to 40). This period was employed to rehearse the scripts, internalize the depicted identity, and enhance recollection of the script parts and improvisation. We achieved this through script internalization and mock interviews focused on enhancing memory and improvisation abilities.

Phase 3 (Days 41 to 60). During this phase, we engaged in practical applications of the SP scenarios in real environments. This was accomplished via comprehensive mock interviews in the classroom and practice sessions in the field (Dry runs).

Logistics for training

Accessories suggested for training SPs included:

- Large conference room with chairs and space to break out into groups for the duration of training (with an additional space with several, small rooms was used for conducting individual mock interviews with visiting or play doctors)
- Printer and printer paper
- Pens
- Small notebooks for SPs
- Watches to note time (otherwise, personal mobile devices can be used)

Training Activities

The following are training activities with short descriptions of what a training team incorporated, focused on, and looked out for.

Phase 1 activities

During the initial phase (days 1-20), we began offering individualized feedback. The training team's activity checklist is as follows:

a. Introduction

- Presented the project to the trainees

- Establish explicit expectations – A greater number of SPs were trained than those who would ultimately be deployed in the field; we clarified and informed them of the criteria for selection.

b. Script and exit interview educational activities.

- Presented the scripts:

• Each SP reviewed the initial page of the script.

We addressed the inquiries and elucidated any elements that were not clearly comprehended by SP.

• Enumerated the things that SPs should address in negotiations regarding the script.

-Instructed on the scripts according to the case.

• Facilitated small group exercises that prompted the SPs to absorb elements of the scripts, including discussing the specific age of the case, their origin, and their job. The learners were instructed to, "Close your eyes and visualize your case." What were they wearing? What kind of footwear were they donning? Develop the individual without experiencing illness. What was their location and the purpose of their being there? What were their thoughts?.

- Outline the daily activities — What actions would this individual undertake each day?

Relevant inquiries were posed such as:
What actions do they take when they feel unwell?
What indicated to them that anything was amiss?
What prompted their decision to visit the clinic?
- Instruct on the introductory statement and inquiries:
• SPs reviewed the opening statement and inquiries. Supervisors guide each group by maintaining records of recommendations from the trainees. This was an opportunity to address any elements of the script that may be absent yet significant to incorporate.
- Validate the translations:
• Translated the script while vocalizing it.
We translated the screenplay in accordance with the specifications of the SPs and had them recite it aloud.
- Facilitated a script comprehension icebreaker:
We requested one of the SP recruits to begin the narrative in the script, and after one line, the subsequent SP would continue the story with an additional sentence. Adhere to this pattern until the script is finalized.
- Executed an initial simulation with an actual clinician or other HCPs. Upon the conclusion of phase 1, each SP engaged in a one-on-one session with a clinician from the advisory group.
-At this juncture, many sub-activities were present:
The clinician commenced the collection of vital signs (e.g., blood pressure, pulse oximetry) as the subsequent phase of health screening.
c. Preliminary internalization of the character - Emulate authentic situations with the SP characters
d. Risk mitigation strategies
From Days 1 to 20, the SPs were initiated into training on risk mitigation measures, which would be reinforced in the subsequent phase 2 (Days 21 to 40). Presented below is a compilation of incidents that may have jeopardized the SP, along by statements to circumvent.
- Injections
• I have an allergy to injections.
• I am currently on other meds.
• I do not receive injections. They induce significant discomfort in me.
•I am quite afraid. I request that you refrain from administering an injection.
• I lack the financial resources to cover this expense, doctor.
I believe this is unnecessary for me. Could you provide further details for my consideration?
- Tablets
•I am experiencing nausea and feel on the verge of vomiting.
• I have not consumed food since yesterday night.
•I have recently consumed additional medications. Allow me to take your medications home, and I will administer them later.
• I shall await my return home. I wish to avoid experiencing dizziness.
• The SP could also feign ingestion of the medicines while the provider is not observing.
- Syrups
• I am experiencing nausea and anticipate vomiting.
• I lack the financial resources to cover this expense, doctor.
- Blood pricks
• I have a severe reaction to pricks.
• I experience significant bleeding. Another physician said that I had a coagulation issue.
• I lack the financial resources to cover this expense, doctor.
- Blood draw
• I am currently unprepared to proceed with this.
• I wish to contemplate this.

•I must confer with my spouse over this matter to ascertain its acceptability.
- Intravenous infusions.
• My previous experience with a needle resulted in an adverse reaction.

Near the conclusion of phase 1, the trainees were informed that they may be engaging with providers using voice recorders. The aims of the audio recordings were elucidated to the SPs. The goals are to:

● Assist the project team in verifying field occurrences and comparing them with the exit interview results.
● Review recorded mock sessions to facilitate learning and adjustments.
● To assess retention, particularly for programs with extended fieldwork duration.

Phase 2 activities

Between days 21 and 40, supervisors transitioned from coaches to their supervisory roles. As the trainees start phase 2 with a solid understanding of their cases and scripts, we did not retain them in a case-specific manner. A transition occurred from acquiring knowledge of case-specific conditions and scripts to being placed in many scenarios and learning appropriate responses. Consequently, we conduct mock interviews during this period, enabling the SP trainees to confront the spectrum of experiences they could meet in a provider's office.

During these moments, we reminded SP that they were the character who were worried about their health and proceeded in character regardless of what happens at the health facility, as long as their actions do not put them (or others) at risk for danger or detection.

Phase 2 activity checklist for the training team is as follows:

a. Increased the complex mock interviews

-Rotate among the SP trainee groups – Supervisors and other members rotated across the case groups and take turns leading each group. This prevented the supervisors from getting too attached to the trainees in their group, but also allowed them to learn the different cases.

- Advised trainees to come dressed as the SP case – This would help them internalize the character and provide an opportunity to hold a group discussion on what would be within the range of appropriate clothing and dress for the case descriptions.

Excerpt from Details Matter on SP clothing and dress

What type of attire would the SPs done, and what did their body language convey? Let us start with the urban setting. We purchased apparel from local marketplaces or from street vendors established by local businesses. The most effective method to prevent errors in this context is to ensure that the SP is attired in his customary clothing. The concept was that the SP should integrate with the local surroundings. The attire for the woman not necessitated innovative thought; we adorned the thirty-year-old in traditional salwar, kameez, and dupatta, complemented with little jewelry. A few glass bangles impart a sense of local culture.

b. Mixed-case group sessions

• SP recruits were randomly assigned to different groups of 6-7 individuals. Individuals take turns: (i) presenting the case, (ii) being the doctor, and (iii) observing and giving feedback.

c. Run through scenarios that SPs may encounter at the facilities, such as:

• No change available when paying consultation fees
• Bribing – The pharmacy person could have asked the SP for a bribe.
• Doctor on the phone – The doctor’s phone rings, and it sounds like he is fighting with his spouse.
• At this stage, we trained the SPs by showing videos of clinical visits to see if the SP recruits could articulate opinions on the clinical setting. Here, by showing the video, the point is to see the opinion of the individual. For example, opinion that SPs have given were of the following kinds: “The doctor was good – he gave an injection – injections cure much more quickly than oral medicines” etc.

d. Coached SP trainees not to fall out of character: During the interaction, an SP cannot be overconfident (e.g., the SP cannot pretend to be a know-it-all and begin offering unsolicited information to the provider, or show the provider that she knows a lot about the disease).

• SPs were be trained to be nonjudgmental of the providers and any health facility staff during all interaction.
e. Memorize the exit interviews – The purpose of memorizing the exit interviews is to ensure SPs begin practicing how to correctly and completely recall aspects of the clinic visit found on the exit questionnaire.
• The training team encouraged the SPs to remember exit interview questions in the order in which they will be asked during data collection. Rehearsing the order of the sections and the questions will help memorization.
• Depending on the structure of fieldwork, some aspects of the questionnaire was not necessary for the SP trainees to memorize.
f. Administered tests on material covered during Phase 1.
g. Conducted mock interviews in different areas: Stage interactions in distracting environments, such as in front of people setting up lunch or outside in a parking lot where there were pedestrians.
• To test distractibility while conducting mocks, supervisors:
o Answered their phone and had a full conversation
o Started some videos on mobile showing traffic with noises
h. Assessed audio recordings
• The best way to assess trainees on recall is to conduct mock interviews with audio recordings, followed by a full exit interview debrief.

Phase 3 activities

The final round of training commenced with simulated interviews, enabling SPs to refine their recollection and improvisation skills. The phase concludes with dry runs, enabling SPs to apply all they have learned in settings that closely resemble actual SP interactions. The dry runs provide an opportunity to observe the sorts of medications, injections, and blood tests that are often administered. The activities checklist for the training team from days 41 to 60 is as follows:
a. Dry runs-We identified providers and facilities where SP trainees performed dry runs; the providers utilized for these dry runs are distinct from those recruited or sampled for the actual fieldwork.
- Assigned SP trainees into pairs (recommended size) - Each pair consisted of one SP and one accompanying person.
-We rehearsed the arrival process via transportation prior to reaching the facility. During the dry runs, the team was responsible for refining the protocols for arriving at the provider clinic using local bus stations or public/private transport.
- Provided directives to SPs prior to their arrival at the facility – SPs received explicit guidance on their further destinations following their visit to the health facility.
- Sustained continuous communication with the SP trainees throughout the day, facilitated by supervisors overseeing specific subsets of the trainees.
b. Debrief sessions for dry runs - We schedule a meeting with SP trainees returning to the training facility, as they may experience prolonged wait times, traffic congestion, transit challenges, and limited consultation periods with providers during dry runs. These are most probable to manifest during dry runs (albeit they will enhance as fieldwork progresses), and it was beneficial to inform the trainees of these potentialities.
Debrief collectively - Debriefing meetings were structured to take place as a broad assembly, enabling SP trainees, trainers, and the project team to gain insights from shared experiences, ensuring that all participants were present to address field issues or ambiguous procedures. The following elements were evaluated during dry runs:
1. Completing exit questionnaires — Experienced SPs followed another SP who presented cases and completed the exit questionnaires for dry runs.
2. Iterative questionnaire design — We utilized dry runs to iteratively develop surveys in real-time.
3. Refresher training — In our initiatives, a cohort of SPs has previously completed a two-month training

program, including periods of fieldwork, but may encounter brief interruptions in operations. In these circumstances, dry runs might function as a refresher.
